# Are Nutraceuticals Effective in COVID-19 and Post-COVID Prevention and Treatment?

**DOI:** 10.3390/foods11182884

**Published:** 2022-09-17

**Authors:** Alessia Catalano, Domenico Iacopetta, Jessica Ceramella, Azzurra Chiara De Maio, Giovanna Basile, Federica Giuzio, Maria Grazia Bonomo, Stefano Aquaro, Thomas J. Walsh, Maria Stefania Sinicropi, Carmela Saturnino, Athina Geronikaki, Giovanni Salzano

**Affiliations:** 1 Department of Pharmacy-Drug Sciences, University of Bari “Aldo Moro”, 70126 Bari, Italy; 2 Department of Pharmacy, Health and Nutritional Sciences, University of Calabria, 87036 Arcavacata di Rende, Italy; 3 Department of Science, University of Basilicata, 85100 Potenza, Italy; 4 Center for Innovative Therapeutics and Diagnostics, Richmond, VA 23223, USA; 5 School of Pharmacy, Aristotle University of Thessaloniki, 54124 Thessaloniki, Greece

**Keywords:** long-COVID, SARS-CoV-2, vitamins, flavonoids, zinc, quercetin, natural bioactive compounds

## Abstract

The beginning of the end or the end of the beginning? After two years mastered by coronavirus disease 19 (COVID-19) pandemic, we are now witnessing a turnaround. The reduction of severe cases and deaths from COVID-19 led to increasing importance of a new disease called post-COVID syndrome. The term post-COVID is used to indicate permanency of symptoms in patients who have recovered from severe acute respiratory syndrome coronavirus 2 (SARS-CoV-2) infection. Immune, antiviral, antimicrobial therapies, as well as ozone therapy have been used to treat COVID-19 disease. Vaccines have then become available and administered worldwide to prevent the insurgence of the disease. However, the pandemic is not over yet at all given the emergence of new omicron variants. New therapeutic strategies are urgently needed. In this view, great interest was found in nutraceutical products, including vitamins (C, D, and E), minerals (zinc), melatonin, probiotics, flavonoids (quercetin), and curcumin. This review summarizes the role of nutraceuticals in the prevention and/or treatment of COVID-19 disease and post-COVID syndrome.

## 1. Introduction

COVID-19, namely coronavirus disease, is today’s most infectious disease caused by severe acute respiratory syndrome coronavirus 2 (SARS-CoV-2). The virus is an enveloped, single-stranded, positive-sense ribonucleic acid (RNA) viruses and belongs to the family Coronaviridae (subfamily Coronavirinae) [1]. It was first detected on 12 December 2019 in Wuhan City, Hubei Province, China [2]. Since then, it quickly spread to other countries around the world, becoming a threat to global health [3]. The pandemic breakout has attained worrisome proportions, stunning national healthcare systems into inaction and necessitating worldwide deployment. Its alarmingly quick transmission and a considerable percentage of morbidity and mortality led the World Health Organization to recognize it as a pandemic on 11 March 2020 [4]. Globally, on 5 August 2022, the WHO reported that there had been 579,092,623 confirmed cases of COVID-19, including 6,407,556 deaths. A total of 12,308,330,588 vaccine doses were reported to have been administered as of 2 August 2022 [5]. COVID-19 is now accepted as a multi-organ disease characterized by a wide spectrum of manifestations. The first variants of COVID-19 [6] led to fever, dry cough, fatigue, and myalgia, and for some, bacterial superinfection in most cases of COVID-19 patients. In severe cases, symptoms progress to COVID-19-associated acute respiratory distress syndrome (CARDS) and respiratory failure, requiring intensive care unit (ICU)-level care. After the COVID-19 era, which is not finished at all yet [7], with new variants emerging (including BA.4 and BA.5 omicron) [8,9], we have now entered the post-COVID era. Fortunately, today, in most cases, COVID-19 has become very similar to a flu-like illness. Mainly, the hassle of quarantine remains as well as the fear of infecting weak people, those with previous pathologies (or diseases), or people who are not vaccinated. What is most worrisome is what will happen next; indeed, many individuals who healed from COVID-19 have developed persistent or new symptoms continuing for weeks or months: this condition is called “post-COVID syndrome” [10]. The number of people that suffer from symptoms after SARS-CoV-2 infection is dramatically increasing. They describe a myriad of symptoms: neurocognitive post-COVID symptoms, such as vertigo, loss of attention, confusion, and brain fog; autonomic symptoms, including chest discomfort, tachycardia, and palpitations [11]; gastrointestinal symptoms, such as stomachache, vomiting, and diarrhea; respiratory symptoms, including tiredness, breathlessness, cough, and throat pain; musculoskeletal symptoms, such as myopathy and arthralgia; psychological-related symptoms, such as post-traumatic stress, insomnia, depression, and anxiety; and other manifestations, including skin rashes, asthenia, anosmia, and parosmia [12]. Post-COVID syndrome is progressively accepted as a novel clinical body in relation to SARS-CoV-2 infection and has been defined as a second pandemic [13]. This disease is not easy to study since several symptoms of post-COVID can be related to exposure to other infective agents or to a devastating circumstance, such as the current pandemic, and be connected with other reasons, including lockdown, unemployment, distress, and fear. Furthermore, in most cases, it is very difficult to trace the variant responsible for the onset of the disease also because post-COVID syndrome becomes evident after some time, even long after the disease has ended. Thus, it is difficult to decide if “possible” and “probable” cases must be considered as post-COVID symptoms. Moreover, post-COVID syndrome may differ on the basis of the variant of COVID-19 that determined the disease. Fortunately, the predominance of the post-omicron COVID-19 condition is lower than the one related to the other strains [14]. Given the number and heterogenicity of symptoms attributable to post-COVID syndrome and the emergence of new variants, including the most recent BA.4 and BA.5 [15], several studies are still addressing to the prevention and treatment of these diseases. Regarding post-COVID syndrome, the first variants usually led to pneumonia-pulmonary fibrosis in post-COVID patients [16]. Now, post-COVID patients complain of asthenia, general fatigue, dyspnea, and weakness. At present, sundry vaccines and drugs are being studied for the prevention and treatment of COVID-19 [17,18]. New therapeutic strategies have also been suggested, including repurposing [19,20], and several research trials are currently in progress. Other non-traditional methods to combat COVID-19 and post-COVID comprise physical exercise [21], yoga and meditation [22], and faster and less expensive methodologies to discover new effective anti-SARS-CoV-2 medicines. Nutraceuticals are known compounds showing immune-boosting, antiviral, antioxidant, and anti-inflammatory activities [23]. They comprehend Zn, vitamins (C, D, and E), curcumin, probiotics, selenium, quercetin, and others [24]. Therefore, their use provides possible alternative preventive and therapeutic support to standard therapies for COVID-19 in adults and children [25,26,27]. Moreover, dietary habits and lifestyle changes may influence the course of the disease [28,29]. Literature research was done on *PubMed/MEDLINE*, *Scopus*, and *Google Scholar* search engines using general keywords such as “post-COVID”, “COVID-19”, “long COVID”, and “nutraceuticals”. All abstracts and full-text articles were examined for their relevance to this review. The aim of this review is to examine the role of nutraceuticals, prebiotics and probiotics, and diet supplementation in the prevention and treatment of SARS-CoV-2 viral infection and post-COVID syndrome.

## 2. Post-COVID Syndrome

**Figure 1:**
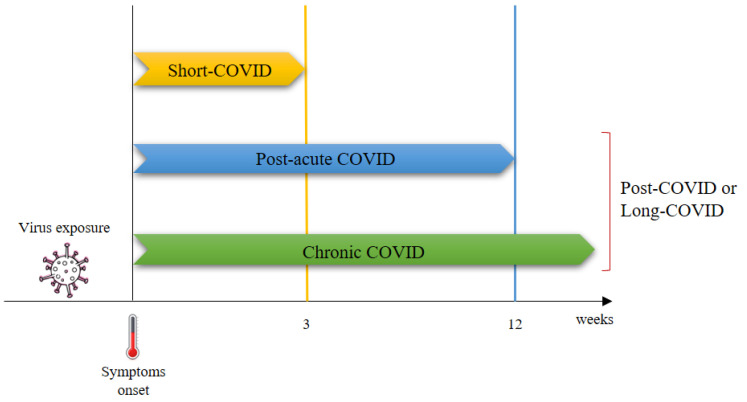
First classification of post-COVID [30].

Currently, there is no universally accepted definition of the post-COVID syndrome. It was defined for the first time by Greenhalgh et al. [30] as a COVID-19-associated illness extending for more than three weeks after the onset of symptoms and chronic COVID-19 as persistent symptoms extending beyond 12 weeks after the onset of symptoms [31]. Depending upon the duration of symptoms, post-COVID or long-COVID syndrome was divided into two stages: post-acute COVID, where symptoms extend to more than 3 weeks but less than 12 weeks, and chronic COVID, where symptoms extend beyond 12 weeks (Figure 1) [30]. 

**Figure 2:**
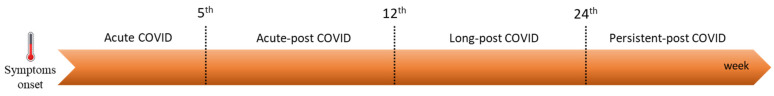
Classification of post-COVID as reported by Fernandez-de-Las-Penas et al. [12].

Then, several studies reported new terms, such as long COVID, Long Haulers, and chronic COVID: thus, a new classification was needed [32]. An integrative classification of post-COVID symptoms was proposed, which include those lasting for more than 24 weeks, and is post-acute COVID (symptoms from 5th to 12th weeks), long post-COVID (symptoms from 12th to 24th weeks), and persistent post-COVID (symptoms continuing for more than 24 weeks) (Figure 2).

The definition of “long-COVID” is not always the same. The terms “post-COVID syndrome”, “long COVID”, or “Long Haulers” are sometimes used interchangeably to mean the same thing [33,34]. However, this is not exactly true. In most cases, long-COVID is used to mean post-acute COVID [35], i.e., post-acute sequelae of COVID-19, a situation characterized by the persistence of COVID-19 symptoms beyond 3 months [36]. However, some authors use the term “long-COVID” to indicate symptoms extending for more 12 weeks from initial symptoms, which is chronic COVID-19 [37]. In our opinion, it is better to refer to the classification of post-COVID to avoid mistakes.

## 3. Nutraceuticals and Dietary Supplements against COVID-19 Disease and Post-COVID Syndrome

**Figure 3:**
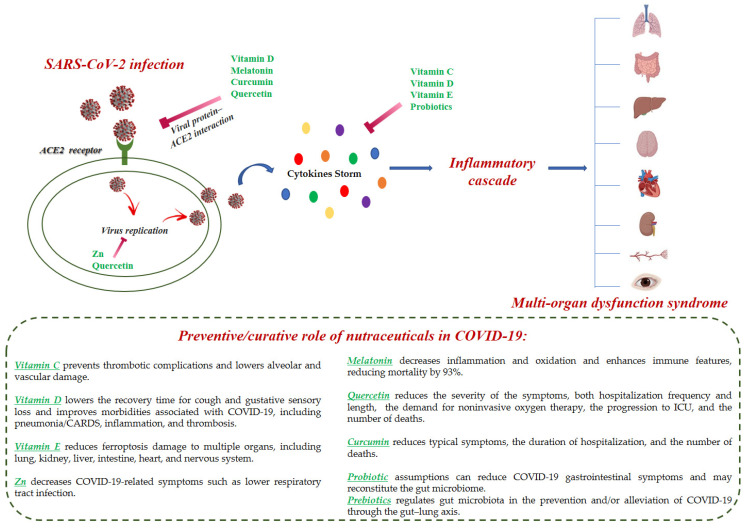
Schematic representation of the molecular events for COVID-19 infection and the preventive/curative role of the nutraceuticals.

The term nutraceutical is a combined terminology for nutrition and pharmaceuticals that popularly reflects the food or its part that has medicinal benefits on health. Nutraceuticals comprise active phytochemicals isolated from plants, dietary supplements, and functional foods with medicinal properties [38,39]. Nutraceuticals have numerous advantages over synthetic drugs, as they are easily accessible and have negligible side effects if administered at the already used and tested dosages. Nutraceuticals include “immune boosting” foods and nutrients, which are those that can regulate the immune system, such as zinc, vitamins, curcumin, resveratrol, and selenium [40]. Considering COVID-19, where there is the absence of efficacious prophylactic and curative drugs and where the mutants of the SARS-CoV-2 spread seriously affect several populations, one of the crucial defenses is a strong immune system. The usual therapies for COVID-19 include antiviral agents, antimicrobials, anti-inflammatories, immunomodulators, angiotensin II receptor blockers, bradykinin B2 receptor antagonists, and corticosteroids [41]. In addition to conventional treatment strategies, the use of nutraceuticals has been considered possibly advantageous in the treatment and/or prevention of COVID-19 and post-COVID-19 [42,43,44,45,46,47]. Higher age, obesity, and weakened immune system along with other diseases, including diabetes mellitus, are recognized risk factors correlated with the severity of COVID-19 disease [48]. For these reasons, the role of nutraceuticals, probiotics, and supplements in decreasing the risk of SARS-CoV-2 infection or attenuating the symptoms of COVID-19 has been largely studied [49,50,51,52]. The use of nutraceuticals for COVID-19 has been often studied in relation to their interaction with angiotensin-2 converting enzyme (ACE2) [53]. The binding between SARS-CoV-2 spike glycoprotein with ACE2 receptor leads to ACE2 downregulation and the resulting enhancement in the level of angiotensin-2 (Ang II) and augmentation of Ang II/Ang II receptor type 1 (AT1R) axis activation that are associated with proinflammatory responses [54]. Consequently, natural compounds that decrease ACE2 activity are useful in the treatment of COVID-19. SARS-CoV-2 utilizes its spike glycoprotein to enter the host cells. The spike glycoprotein receptor-binding domain (RBD) interacts with ACE2 on the host cells. The recent Omicron variant is characterized by 32 mutations in the spike glycoprotein, including 15 of them in the RBD [55]. Numerous molecular modeling docking studies on natural compounds have been carried out to evaluate their anti-ACE2 activity by the preventing RBD–ACE2 interaction [56,57,58]. Laboratory and clinical data support the possible advantages that some bacterial and molecular compounds may determine in the immune response to respiratory viruses and their regulatory role in systemic inflammation or endothelial damage, both representing two essential aspects of COVID-19 [59]. In that regard, the use of probiotics, prebiotics, and postbiotics has been also studied in the fight against SARS-CoV-2 infection [60]. There is a clinical demonstration that the modification of the intestinal microbiota by using these supplements can regulate COVID-19 progression. Some of the main findings were represented by the reduction of the course of the disease and the severity of symptoms [61]. Vitamin C, vitamin D, vitamin E, flavonoids, prebiotics, probiotics, zinc, and melatonin are the principal dietary supplements that are being studied for their use in COVID-19 (Figure 3) [62]. Moreover, recent studies demonstrated that the administration of high doses of vitamins C, D, and E, in addition to omega-3 fatty acids and zinc, may potentially result in a clinical advantage for hospitalized patients [63]. These supplements may reduce the viral load, the disease severity, and hence hospital stay thanks to their immunomodulatory and antioxidant effects. Furthermore, the deficiency of these nutritional substances may be responsible of improved susceptibility to infections and dysfunction of the immune system. Nevertheless, there are no specific randomized controlled trials (RCTs) on vitamin supplementation in the prevention or treatment of COVID-19 infection. Therefore, clinical trials are needed to confirm the role of these supplements in COVID-19 prevention and treatment [64,65]. The use of nutraceuticals in post-COVID syndrome is currently under study. Several reports are described in adults [66,67], older people [68], and children [69,70] for different symptoms including olfactory loss [71], telogen effluvium [72], and depression and anxiety [73]. 

### 3.1. Vitamins

Vitamins are micronutrients that occupy a crucial position in the appropriate organization and function of proteins and in some physiological processes and signal pathways. The term “micronutrients” is related to the fact that they are required in small, generally microgram amounts daily. However, in critical illness, the requirements for these micronutrients can be significantly increased [74]. Their usefulness in COVID-19 patients has been demonstrated and recent research addressed the investigation of their mechanism of action [75].

#### 3.1.1. Vitamin C

**Figure 4:**
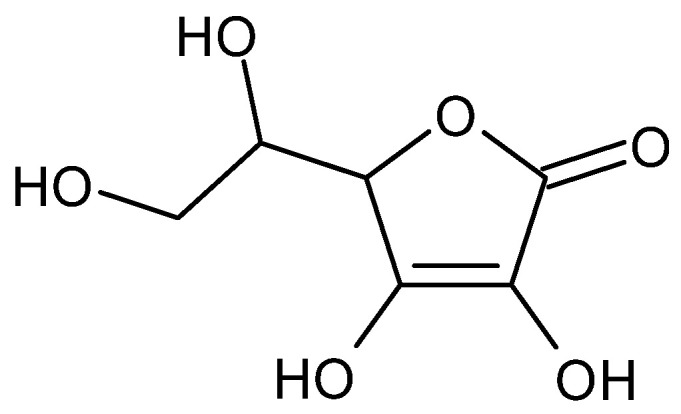
Structure of vitamin C.

Vitamin C, also known as ascorbic acid (Figure 4), is a powerful molecule with pleiotropic functions. It has been demonstrated to perform an essential role in immune function; it shows antioxidant, antiviral, anticancer, and antithrombotic effects [76]. It is mostly represented in the plant world (spinach, chard, broccoli, sweet potatoes, cauliflower, pepper, carrots, peppers, and others) [77,78]. Many fruits are also rich in vitamin C, such as oranges, strawberries, apples, cherries, and particularly kiwi. The levels in the Hayward green cultivar are typically between 80 and 120 mg per 100 g fresh weight [79,80].

At pharmacological doses, vitamin C is beneficial to patients affected by CARDS and other respiratory illnesses. Moreover, high-dose intravenous vitamin C (HDIVC) may be advantageous in patients with diverse viral diseases [81]. The antiviral activity of vitamin C is due to the support of lymphocyte activity, enhancement of interferon-α production, and modulation of cytokines, which lowers inflammation, improving endothelial dysfunction and restoring mitochondrial function. A high dose of vitamin C may have a virucidal effect since it inhibits viral growth in vitro [82]. The current statement describes the possible use of vitamin C as a prevention or treatment of COVID-19 patients [83,84]. Its use has been suggested used as a primary preventative measure for susceptible populations such as the elderly, those suffering from comorbidities, and healthcare workers with higher exposure risks [85]. Vitamin C can suppress the cytokine storm, reduce oxidative stress, decrease inflammation, prevent thrombotic complications, and lower alveolar and vascular damage [86]. However, the negative effect of vitamin C is that it can lead to the formation of urinary stones or the occurrence of nephropathies [87]. The recently reported randomized trial in sepsis demonstrated poorer outcomes in those patients receiving vitamin C [88]. Other studies are ongoing or have recently finished in assessment of the role of vitamin C in the treatment of COVID-19 (Table 1). A double-blind RCT was carried out by Majidi et al. (2021) [48] in critically ill COVID-19 patients. The daily supplementation of 500 mg vitamin C for 2 weeks markedly enhanced the survival of COVID-19 patients during the post-supplementation period. Moreover, the authors demonstrated that there was no side effect on kidney function, arterial blood gas parameters, and electrolytes (sodium, calcium, and phosphorus) after vitamin C supplementation. The treatment with HDIVC of CARDS and multiorgan dysfunction related to COVID-19 had been previously hypothesized by Liu et al. (2020) [89]. The authors predicted that HDIVC could suppress cytokine storm caused by COVID-19, help to enhance pulmonary function, and lower mortality for patients with COVID-19. In the RCT study, they found that HDIVC was beneficial in terms of stability, availability, safety, and cost compared with other treatments. Zhao et al. (2021) [90], in a retrospective case series study, assessed the beneficial effects of HDIVC in patients with severe COVID-19 pneumonia. The study was carried out on twelve patients (six severe and six critical). All patients received HDIVC (mean 163 mg/kg in severe patients). Significant improvements were obtained in C-reactive protein (CRP), lymphocyte count, and CD4. After HDIVC therapy, the improvements in severe patients were higher than those in critical ones. HDIVC (11 g per day average for a 70 kg person) was shown to be advantageous in terms of inflammatory response and immune and organ function. In a retrospective before–after case-matched study, Zhao et al. (2021) [91] studied the treatment of 55 patients with moderate COVID-19 with an HDIVC protocol of 100 mg/kg/day for seven days from admission; the control group was not treated with HDIVC. There was a substantial decrease in the number of patients treated with HDIVC that evolved from moderate to severe disease (*p* = 0.03). In addition, during the first week, there was a reduced systemic inflammatory response syndrome (SIRS) duration and occurrence (*p* = 0.0004 and *p* = 0.0086, respectively) in the treated patients with respect to the control group. A recent placebo-controlled pilot study by Zhang et al. (2021) [92], carried out in three hospitals located in Hubei, China, in 56 critically ill COVID-19 patients, evidenced a substantially lowered mortality after HDIVC treatment (daily dose of 24 g of ascorbate). HIDIVC did not affect ventilation-free days but possibly provided a possible sign of benefit in oxygenation for critically ill patients with COVID-19, characterized by an enhancement in PaO_2_/FiO_2_ ratio.

#### 3.1.2. Vitamin D

**Figure 5:**
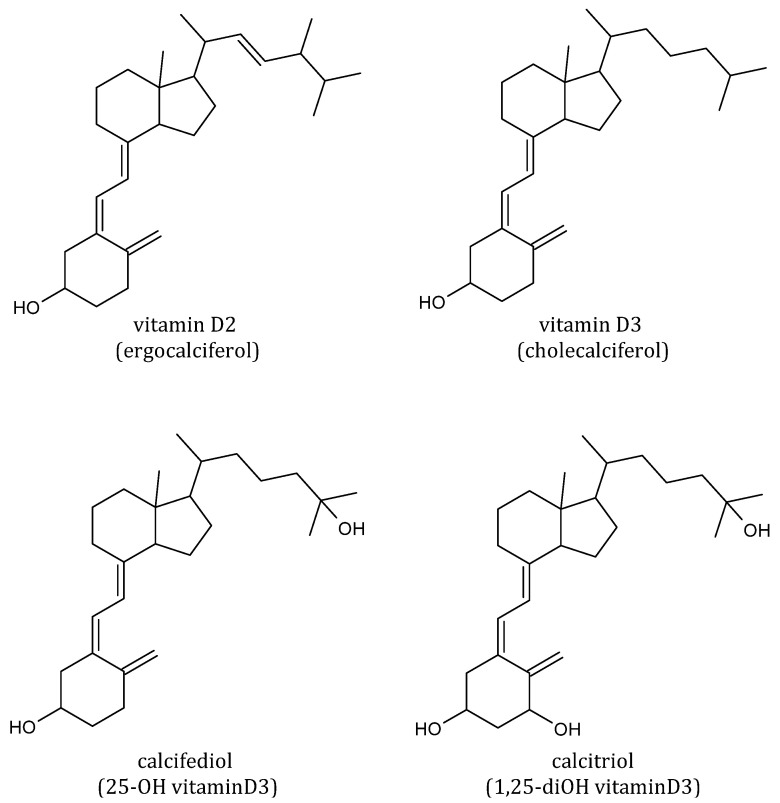
Structures of vitamin D2, D3, and its main metabolites.

In the past decade, the crucial function of vitamin D in inflammation and immunomodulation was progressively recognized [93]. The preventive and therapeutic contribution of vitamin D in COVID-19 has been widely studied [94,95,96]. Recently, clinical trials and meta-analysis studies regarding the role of vitamin D in the prevention, progression, and severity of prevention of COVID-19 infection, have been reported [97,98]. Vitamin D deficiency seems to aggravate COVID-19 [99]. The severity of hypovitaminosis D was in relation to the prognosis of COVID-19 since COVID-19 cases in which hypovitaminosis D was also found were more susceptible to experience severe COVID-19 [100]. Moreover, hypovitaminosis D was linked to a higher COVID-19 mortality risk [101]. Besides being a fat-soluble vitamin, vitamin D is also a steroid hormone, playing a vital role in modulating the immune system together with maintaining serum calcium homeostasis [102]. Vitamin D can derive from supplements as vitamin D2 (ergocalciferol) and D3 (cholecalciferol) (Figure 5). Vitamin D is obtained from limited dietary sources such as oily fish and egg yolks or from photochemical and thermal transformation of the cholesterol precursor 7-dehydrocholesterol in skin exposed to ultraviolet B radiation [103]. However, the major source of vitamin D remains sun exposure. The conversion to its hydroxylated metabolites is determined by skin exposure to ultraviolet B (UVB) radiation through the activity of specific hydroxylases. Among these, calcifediol (25-hydroxyvitamin D3) and calcitriol (1,25-dihydroxyvitamin D3) are the immunologically active forms.

Vitamin D acts by modulating the renin-angiotensin system (RAS) and the expression of the ACE2; thus, it may prevent adverse. COVID-19 outcomes [104]. It mitigates lipoprotein (LPS)-induced acute lung injury by inducing the ACE2/Ang 1–7 axis and suppressing the ACE/Ang II/AT1R axis [105]. Moreover, vitamin D acts on different mechanisms of the immune system to restrain the virus, including reduction of the entry of the virus, lowering the levels of pro-inflammatory cytokines and enhancing the anti-inflammatory ones, increasing the development of natural antimicrobial peptide, and activating defensive mechanisms including macrophages that are able to demolish SARS-CoV-2 [106,107]. Accordingly, there is a growing amount of data that shows the association between serum calcifediol and the different clinical outcomes of SARS-CoV-2 infection [108]. A preliminary study showed the inhibitory effect of calcitriol on the nasal epithelium infected with the virus [109]. In a genomic study, Glinsky (2020) suggested that, in addition to inhibiting the expression of ACE2, vitamin D is also able to disrupt the function of SARS-CoV-2 gene proteins, acting as a repressor of ACE2 expression [110]. Annweiler et al. (2020) [104] hypothesized that supplementation with high-dose vitamin D could ameliorate the prognosis of COVID-19 in high-risk older patients. The first reports showed that cases with COVID-19 had on average significantly lower calcifediol levels compared with negative patients [111]. Likewise, a substantial reverse relationship was found in 20 European countries between the mean serum concentrations of calcifediol and the number of COVID-19 cases and mortality [112]. A RCT study showed that vitamin D3 supplementation (5000 IU daily for 2 weeks) lowers the recovery time for cough and gustative sensory loss in patients with sub-optimal vitamin D status and showing mild to moderate symptoms of COVID-19; this was also demonstrated from the progressive decrease in BMI and IL-6 levels [113]. A randomized prospective open-label study carried out in India on 87 patients with COVID-19 and hypovitaminosis D also showed that supplementation with vitamin D to standard care significantly ameliorated inflammatory markers. Patients receiving 60,000 IU of daily supplemental vitamin D for eight days showed an improvement in levels of C-reactive protein, lactase dehydrogenase, IL-6, ferritin, as well as neutrophil to lymphocyte ratios [114]. Further studies showed that the assumption of vitamin D 200,000–300,000 IU bolus and then reduction to a maintenance dose reduces the risk of contracting COVID-19 and the severity of the disease [115]. The issues and morbidities associated with COVID-19, including pneumonia/CARDS, inflammation, and thrombosis, may be improved by vitamin D [116]. Furthermore, severe-COVID-19 patients are often predisposed to bone fragility and osteoporosis, which can be related to vitamin D deficiency and altered platelet-related parameters. Thus, the influence of the relationship between vitamin D and PLT on the risk and outcome of COVID-19 disease has been studied [117]. Moreover, hypovitaminosis D was associated with a greater COVID-19 mortality risk [118]. Most of the abovementioned reasons and results reinforce the use of supplementation with vitamin D as potential prophylaxis for COVID-19. Recently, it has been speculated that vitamin D may play a complementary role in the development of vaccine efficacy [119]. Actually, vitamin D deficiency (calcifediol below 50 nmol/L) is still widespread despite its important role [120].

#### 3.1.3. Vitamin E

Supplementation with foods that are a source of vitamin E has been used to control nutritional deficiencies and obesity and promote acceptable nutritional status in COVID-19 patients by improving immune and antioxidant activities during the infectious phase [121]. The body is not able to synthesize vitamin E. It exclusively formed by photosynthetic processes of plants and therefore must be consumed from outside sources in minor quantities. It is found in abundance in sunflower oils and olive as well as nuts, soybeans, avocados, wheat, and green leafy vegetables [122]. Vitamin E comprises a class of liposoluble compounds, including tocopherols and tocotrienols, characterized by a hydroxylated chromanol ring attached to a hydrophobic phythyl side chain. Despite the existence of multiple tocopherol and tocotrienol vitamers, the attribute of “vitamin” is only given to α-tocopherol [123]. α-TOH is a lipid-soluble antioxidant that serves for the preservation of cell membranes, as it acts as a defense against oxidative stress [124]. It traps reactive oxygen species, thus preventing the oxidative explosion associated with SARS-CoV-2 [125]. Vitamin E is able to reduce inflammatory cytokine production, promote T-cell proliferation and differentiation, and influence inflammatory responses in various tissues, including the lungs [126]. In animal and human models, vitamin E ameliorates the immune response by decreasing the production of nitrogen oxide, thus downregulating prostaglandin E2 and inhibiting cyclooxygenase-2, initiating T-lymphocyte signals, and modulating Th1/Th2 balance. Furthermore, it acts as an immunoregulator through protein kinase C regulation [127]. Investigations of the effectiveness of vitamin E in COVID-19 are still underway. However, its immunomodulatory and preventive function from the oxidative disruption have contributed to its recognition as a possible supplement for COVID-19 [127]. Vitamin E supplementation at a high dose of 500 mg/kg can also act as a therapeutic drug to inhibit ferroptosis, which is one of the central mechanisms of programmed cell death in COVID-19 patients, and reduce ferroptosis damage to multiple organs, including lung, kidney, liver, intestine, heart, and nervous system [128,129]. The use of vitamin E has been also studied in vulnerable populations including the elderly and pregnant women, conditions in which the effect of COVID-19 infection is specifically dangerous for health [130]. In pregnancy, important alterations occur in the immune, hematological, cardiovascular, and respiratory systems. As COVID-19 mainly affects these systems, doctors have concerns regarding COVID-19’s influence on pregnant women. In effect, COVID-19 may cause obstetric complications such as miscarriage, preterm labor, pre-eclampsia, and fetal distress [131]. Increased ROS have been demonstrated as a result of the production of free superoxide radicals and mitochondrial activity of the placenta during pregnancy [132]. As an antioxidant molecule, vitamin E can decrease oxidative stress during pregnancy [133]. A recent study evaluated maternal serum afamin and vitamin E amount in pregnant women with COVID-19 (60 pregnant women with COVID-19 infection and 36 age-matched pregnant women without any defined risk factors) and the association with adverse perinatal outcomes. Afamin is a specific binding pleiotropic glycoprotein for vitamin E, and it is an indicator of oxidative stress. The study in the group of women with COVID-19 showed high levels of afamin and low levels of vitamin E in all trimesters of pregnancy [134].

### 3.2. Zinc

Zinc (Zn) is a transition metal that, after iron, is the second in abundance in the trace metals in the human body. Its crucial role in multiple cellular functions has been recognized, such as the maintenance of immune health, thus playing an essential role in antiviral functions. Zn also acts as an anti-inflammatory, antioxidant, and membrane stabilizer agent. Zinc is present in different foods, mainly in those of animal origin (fish, meat, egg yolk) to those of vegetable origin (wheat germ and oats), legumes, nuts and walnut seeds, pumpkin, and sesame [135,136]. Zn deficiency can lead to lymphopenia, caused by a reduction in the development of lymphocyte B cells in the bone marrow. Coronavirus RNA polymerase activity is inhibited by Zn, thus conferring to this metal a role in COVID-19 disease [63,137]. Zn supplementation determines the improvement in the efficacy of other treatments, such as hydroxychloroquine [138]. Furthermore, a retrospective study carried out on 141 COVID-19 patients showed that the association of Zn with low-dose hydroxychloroquine led to significantly fewer hospitalizations [139]. A case series with high-dose Zn on four COVID-19 patients also showed clinical symptomatic improvements [140]. Zn supplementation may lower decrease COVID-19-related symptoms such as lower respiratory tract infection. Thus far, there is no definite awareness of the amount of Zn required to have a therapeutic effect. Several factors including the presence of pre-existent Zn deficiency and the variance in Zn bioavailability and absorption must be taken in consideration [141]. A RCT study showed the safety and feasibility of intravenous Zn treatment and the ability of administering high-dose intravenous Zn to invert the acute-phase Zn deficiency related to COVID-19 [142]. Further investigation in this field is needed with larger RCTs.

### 3.3. Melatonin

**Figure 6:**
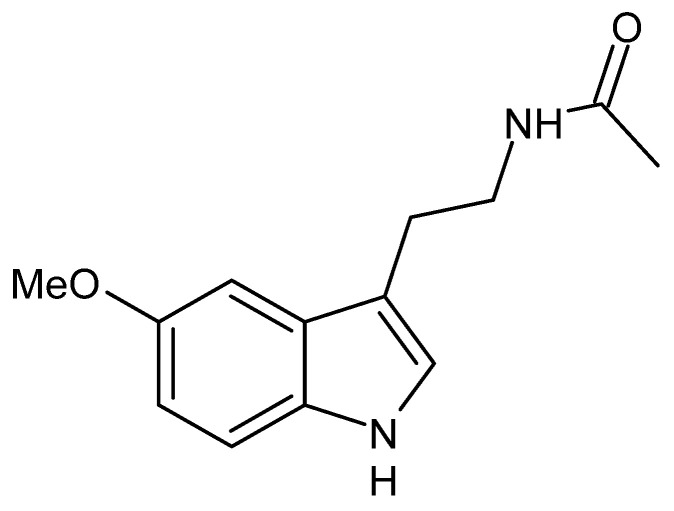
Structure of melatonin.

Melatonin (*N*-acetyl-5-methoxytryptamine, Figure 6) is a hormone secreted by the pineal gland mainly during the night and more in infants and adolescents than in the elderly.

Melatonin is important in the regulation of other hormones and maintenance of the body’s circadian rhythm. Melatonin is substantially implicated in psycho-neuroendocrine immunology (PNEI), aging mechanisms, and stress management; moreover, it interacts with cortisol and immunity and inflammasome pathways, interfering with COVID-19 [121]. The combination of melatonin with conventional therapies may substantially reduce the mortality of severely ill COVID-19 patients. The main target of melatonin is the immune system; thus, it has a protective action against gene-mutated variants [143]. Moreover, melatonin does not act as a virucidal but shows indirect antiviral action by reducing inflammation, oxidation, and enhancing immune features. Anti-inflammatory effects may be related to sirtuin-1 (SIRT-1) and nuclear factor kappa-B (NF-κB). Its anti-oxidative effect is due to up-regulation of anti-oxidative enzymes such as superoxide dismutase, down-regulation of pro-oxidative enzymes including nitric oxide synthase, and interaction with free radicals. Furthermore, melatonin improves proliferation and maturation of natural killing cells, T and B lymphocytes, and monocytes and granulocytes in bone marrow and other tissues [144]. The direct inhibitory effects of melatonin on the entry of the SARS-CoV-2 virus into the human host have recently been explored [145]. Melatonin may bind SARS-CoV-2 RBD and ACE 2, exerting a dual-binding effect. Melatonin is also a significant inhibitor of calmodulin, which is required for the activation of ACE2. Thus, its prolonged effect on ACE2 may be due to its binding to the receptor and the inhibition of calmodulin [131]. It also significantly inhibits inflammasome stimulation that may indirectly diminish the severity of the cytokine storm and lung destruction [146]. Furthermore, it can restore the circadian rhythm and mitochondrial metabolism, serving as an adjuvant in COVID 19 [147,148]. Recently, Hasan et al. (2021) [149] completed a single-center, prospective RCT on the protective effect of melatonin in severe-COVID-19 patients. The protocol consisted of standard therapy with oxygen intubation, remdesivir, levofloxacin, dexamethasone, and enoxaparin. Half of the patients additionally received 10 mg of melatonin. Interestingly, the mortality rate was 17.1% in the conventional therapy group and 1.2% in melatonin group; thus, overall, the mortality was reduced by 93% in patients treated with melatonin.

### 3.4. Flavonoids

Flavonoids are a wide class of nutraceuticals present in numerous foods and vegetables in the diet, having precious pharmacological properties including antioxidant, antitumor, and anti-inflammatory effects [150,151]. Several flavonoids have been also studied for their antiviral properties in vitro and in vivo [152]. Flavonoids determine the inhibition of viral protease, RNA polymerase and mRNA, virus replication, and infectivity, thus suggesting an antiviral activity [153]. Moreover, they have shown anti-viral and immunomodulatory activities against coronaviruses [154]. Nowadays, flavonoids are a source of possible agents for COVID-19 [155]. Flavonoids may target several inflammatory pathways associated with SARS-CoV-2, such as modulating NOD-like receptor protein 3 (NLRP3) inflammasome, toll-like receptors (TLRs), bromodomain-containing protein 4 (BRD4), nuclear factor erythroid-derived 2-related factor 2 (NRF2), and ACE2 [156]. Furthermore, they can modulate the immune system and macrophage profile and natural killer cells and increase anti-inflammatory mechanisms [157]. An instructive molecular modeling study revealed that epicatechin from *Hypericum perforatum* provided a better inhibition of the ACE2 with favorable pharmacokinetic properties than the other known ACE2 inhibiting compounds [158]. Another study showed that the citrus flavonoid naringin is able to inhibit ACE2 enzyme, showing very low estimated docking energy (−6.85 kcal/mol) [159]. Several flavonoids, including apigenin, fisetin, luteolin, kaempferol, jusanin, and quercetin, have been effectively used for the prevention and/or treatment of COVID-19 [160,161] and post-COVID [162,163,164].

#### Quercetin

**Figure 7:**
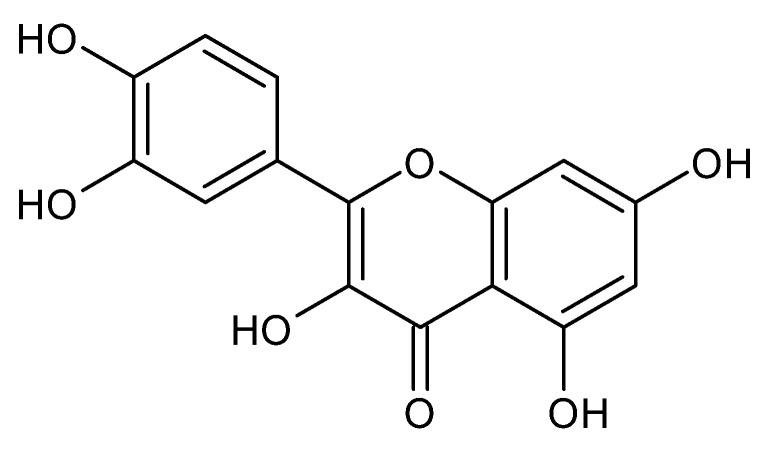
Structure of quercetin.

Quercetin (3,3′,4′5,7-pentahydroxyflavone, Figure 7) is a large, distributed plant flavonoid present in vegetables, leaves, seeds, and grains [165].

It shows antioxidant, anti-inflammatory, anti-cancer, and immunoprotective effects, and it can prevent many chronic diseases. It also inhibits lipid peroxidation, platelet aggregation, and capillary permeability and promotes mitochondrial biogenesis. Furthermore, quercetin has been studied for its promising antiviral effects due to its ability to the inhibition of proteases, polymerases, reverse transcriptase, DNA gyrases, and binding viral capsid proteins [166]. The phytochemical quercetin supplementation, in the form of foods or nutraceuticals, may help in the prevention of COVID-19 through multiple mechanisms [167]. Computational studies have highlighted that quercetin is able to inhibit the major targets of SARS-CoV-2 including 3-chymotrypsin-like protease, 3CLpro, and papain-like protease; PLpro, which are two enzymes essential for the replication of the virus and therefore important drug targets [168]; and RNA-dependent RNA polymerase and spike (S) protein [169]. Nguyen et al. (2012) [170] showed that quercetin inhibited recombinant SARS-CoV 3CLpro activity by up to 80%. Abian et al. (2020) demonstrated that this activity was related to the destabilization of its structure [171]. The docking binding energy for 3CLpro and PLpro correspond to −6.25 and −4.62 kcal/mol, respectively, as assessed by molecular modeling studies [172]. Furthermore, quercetin has additional activities uniquely aimed at counteracting SARS-CoV-2. Specifically, it alters the expression of 30% of genes encoding protein targets of SARS-CoV-2 in human cells, and thus, it potentially interferes with the activities of 85% of SARS-CoV-2 proteins [110]. Moreover, quercetin inhibits protein disulfide isomerase (PDI), the enzyme involved in platelet-mediated thrombin formation, and may alleviate coagulation abnormalities that may be found in COVID-19 patients [173]. Finally, it may interact with NLRP3 [174]. These receptors are activated by SARS-CoV-2, leading to a cytokine storm and destructive inflammation and causes ALI/CARDS in COVID-19 patients [175]. Activation or inhibition of the NLRP3 inflammasome is influenced by regulators such as thioredoxin-interacting protein (TXNIP), SIRT1, and NRF2. The anti-inflammatory activity of quercetin is related to the suppression of the NLRP3 inflammasome by acting on these regulators. Additionally, quercetin suppresses inflammation through interference in various signaling pathways, especially NF-κB [176]. With regard to human studies, the interim results from one RCT that demonstrated that quercetin supplementation improved viral clearance and partially lowered symptoms severity [177]. In another RCT, the therapeutic efficacy of quercetin was evaluated in combination with antiviral drugs in hospitalized COVID-19 patients, showing that it diminished the hospitalization period. Moreover, the serum levels of quantitative C-reactive protein (q-CRP), lactate dehydrogenase (LDH), and alkaline phosphatase (ALP) were decreased more efficiently after quercetin administration [178]. Moreover, a pilot, controlled, and open-label RCT assessed that the administration of Quercetin Phytosome® (QP), a novel bioavailable form of quercetin, statistically shortened the timing of conversion of the molecular test from positive to negative and the severity of the symptoms [179]. In a study on 152 COVID-19 outpatients by Di Pierro et al. (2021) [180], the administration of a daily dose of QP for 30 days determined a decrease of both hospitalization frequency and length. The demand for noninvasive oxygen therapy, the progression to ICU, and the number of deaths were also reduced. The combination of quercetin with standard therapy in the first period of viral infection was shown to improve the early symptoms and help in preventing the severity of the progression of COVID-19. Recently, Rondanelli et al. (2022) [181] also evaluated the potential effect of 3 months’ supplementation with QP (250 mg twice a day) as prevention for symptomatic COVID-19. This pilot study, which was carried out on 120 subjects (63 males, 57 females, age 49 ± 12), demonstrated that QP administration determined a 14% higher protection factor of contracting the COVID-19 infection. The results obtained are encouraging, but further studies with a larger number of participants and longer follow-up are needed before considering quercetin for regular prophylaxis of COVID-19.

### 3.5. Curcumin

**Figure 8:**
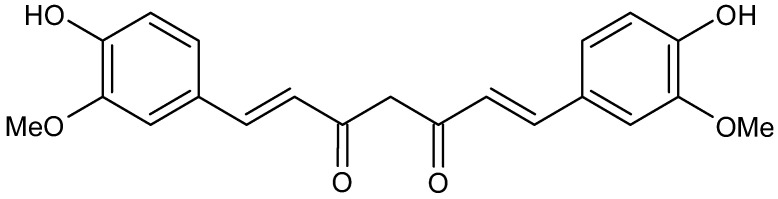
Structure of curcumin.

Curcumin (diferuloylmethane, Figure 8) is the primary curcuminoid derived from the rhizome of turmeric (*Curcuma longa*). It has shown diverse biological functions, such as anti-inflammatory, antioxidant, anticancer, and antimicrobial properties. Besides the antifungal and antibacterial properties, it may also act as an anti-viral compound by inhibiting the replication in a wide range of viruses [182]. Therefore, it was proposed as a potential treatment against COVID-19 [183].

Curcumin is, in fact, an interesting compound to study for management or treatment of COVID-19 thanks to its relative safety and to its broad spectrum of antiviral activity against enveloped viruses, such as SARS-CoV-2. It may directly modify the spike protein and/or ACE2 and induce host antiviral responses by targeting NRF2 and HMGB1 and block NF-κB, inflammasome, HMGB1, and IL-6. Moreover, it inhibits NADPH oxidase; thus, it dampens ROS production and alleviates oxidative tissue injury [184]. Manoharan et al. (2020) [185] indicated curcumin as a “wonder” drug in COVID-19 management for its inhibitory activity exerted by blocking the host viral interaction between viral spike protein and ACE2 receptor at an entry site in humans. It may also actively interfere via modulating the proinflammatory effects of Ang II-AT1 receptor-signaling pathways by lowering respiratory distress in COVID19 treatment. Moreover, they elucidated that the topic application of curcumin as emulsion may efficiently hamper SARS-CoV2 infections in humans considering the localization of the viral entry site of ACE2 receptor in nasal cells, eyes, and the mucosal surface of the respiratory tract. Finally, Liu et al. (2020) [186] suggested curcumin as a therapy against pneumonia and fatal CARDS in humans. Several computational studies underlined the capability of curcumin of interacting with several target proteins of SARS-CoV-2. Shanmugarajan et al. (2020) [187] showed that curcumin inhibits the binding of spike glycoprotein to ACE2 receptor, thereby attenuating the viral infection. Patel et al. (2021) [188] showed that curcumin and its derivatives act as inhibitors of the spike protein displaying binding energies, ΔG, ranging from –10.98 to –5.12 kcal/mol (6CRV) and –10.01 to –5.33 kcal/mol (6M0J). The most interesting compound was bis-demethoxycurcumin, which showed the best binding affinity to the spike protein of SARS-CoV2. Jena et al. (2021) [189] reported the potential of catechin and curcumin to interact with the S protein of SARS-CoV-2 and ACE2 of the human cell membrane and with the RBD/ACE2-complex.

In vitro studies are specified below. Marín-Palma et al. (2021) described the combined effects of curcumin as antiviral and anti-inflammatory against DG614 strain and Delta variant [190]. They demonstrated that 10 µg/mL of curcumin showed an antiviral effect of 99% and 99.8%, respectively, suggesting that this nutrient affects the SARS-CoV-2 replicative cycle and may exhibit a virucidal effect independently from the virus strain/variant. Moreover, the pro-inflammatory cytokines IL-1β, IL-6, and IL-8 released by peripheral blood mononuclear cells triggered by SARS-CoV-2 were reduced after treatment with curcumin. The study by Bormann et al. [191] demonstrated that curcumin potently neutralizes SARS-CoV-2 in Vero E6 and human Calu-3 cells at low, subtoxic concentrations. Further, curcumin treatment significantly lowered SARS-CoV-2 RNA levels in cell culture supernatants. The effectiveness of curcumin on outcomes of hospitalized COVID-19 patients was also recently reviewed [192]: the additive treatment with various formulations of curcumin brought the reductions of typical symptoms, the duration of hospitalization, and the number of deaths. Moreover, this therapy led to improvement in cytokine storm manifestation by reducing pro-inflammatory and stimulating anti-inflammatory pathways. Interestingly, curcumin bioavailability can be enhanced by 2000% using piperine as an adjuvant. A double-blind, controlled RCT indicated that administration of a combination of curcumin and piperine reduced the days of remission of symptoms and the oxygen requirement. Dose-escalating studies have demonstrated the safety of curcumin after 3 months of administration [193].

### 3.6. Prebiotics and Probiotics

Gastrointestinal disturbances are common in COVID-19 patients and may affect the host’s intestinal microbiota: it means that the diversity and population of beneficial bacteria may change [194,195]. Dysbiosis has been widely associated with COVID-19 severity [196]. The reduction of gut microbiota richness persists even six months after recovery following SARS-CoV-2 infection [197]. The modulation of the intestinal microbiota by probiotics, prebiotics, synbiotics, postbiotics, paraprobiotics, and psychobiotics represent a potential additive approach for enhancing health of COVID-19 patients [198,199]. Specific probiotic assumptions can reduce COVID-19 gastrointestinal symptoms, which are often worsened by the use of antibiotics and anti-inflammatories. Probiotics may reconstitute the gut microbiome and consequently modulate the immune system, reduce the vulnerability to infections, and increase the amount of resistance genes [200]. Furthermore, probiotics may modulate other crucial aspects in the severity of COVID-19 cases, such as improving the production of Treg cells to control inflammation [201], reducing D-dimer levels that are involved in coagulopathy [202], and intensifying the immune efficacy of COVID-19 vaccines [203,204]. Nevertheless, more studies are needed to evaluate the effects of probiotics and establish the right doses, the intervention time, and the mechanisms of action in COVID-19 disease. Two important groups of prebiotics are represented by fructo-oligosaccharides and galacto-oligosaccharides, which exist in low quantities in foods and show beneficial effects on human health [205]. Among prebiotics [206], tea polyphenols (TPs) have been shown to regulate gut microbiota in the prevention and/or alleviation of COVID-19 through the gut–lung axis [207]. The gut and lungs have been demonstrated to belong to a shared mucosal immunity that is the gut–lung axis [208,209]. Therefore, adjusting host-microbiota balance in the lung and gut can be a good way to curb COVID-19. Given the ability of probiotics to act as immunomodulators, anti-inflammatories, antioxidants, and antivirals, their use may represent a correct way to support the restoration of the gut microbiota [210]. Probiotics are living micro-organisms that give an advantage to the host’s health if administered in proper doses [211]. Some of their general mechanisms are represented by inhibition of bacterial adherence and invasion capacity in the intestinal epithelium and improvement of the gut barrier function and immune system [212]. Probiotics have been reported to confine the virus entry by restoring the ACE2-containing epithelial barrier. Probiotics may also release ACE-inhibitory peptides that can reduce Ang II expression and induce the synthesis of short-chain fatty acids, which regulate blood pressure and inflammation. They reduce NO production and stress oxidation leading to inflammatory pathways (NLRP3 and NF-κB) downregulation. Bacteriocin and other anti- and pro-inflammatory cytokines produced by the effects of probiotics might balance pro- and anti-inflammatory cytokine levels and enhance T-cell count in the SARS-CoV-2 patients infected. Moreover, probiotics may also reduce hyaluronan synthesis, which could eventually improve CARDS [213]. Some clinical trials regarding probiotics for the management of COVID-19 are already ongoing [214]. Some representative RCTs of probiotic intervention in COVID-19 are summarized in Table 2.

### 3.7. Nano-Nutraceuticals

Nanotechnology is widely used in different fields of research with countless biomedical science applications from cancer nanomedicine [221,222] to antimicrobial activity [218,219], and finally, advancements in the area of nanomedicine in healthcare have been carried out in fighting the COVID-19 pandemic [223,224,225,226,227]. Nanomedicine applications and lipid-based nanoparticles can also aid in the occurrence of efficacious vaccines and/or therapeutics against COVID-19 [228]. Potential immuno-nanomedicine strategies to curb COVID-19 have been also proposed [229]. Recently, nano-nutraceuticals have been suggested to manage pre- and post-COVID infections [230,231,232], including fisetin flavonoid nanoparticles [233], resveratrol and Zn nanoparticles [234], and curcumin nanoparticles [235]. Actually, for example, the clinical use of curcumin is hampered by its low oral bioavailability. The use of several formulations, such as packaging with nanoparticles, liposomes, and micelles, represents a suitable strategy to improve curcumin bioavailability [236]. Sharma et al. reported that curcumin-encapsulated polysaccharide nanoparticles (CUR–PS-NPs) strongly inhibit the release of cytokines, chemokines, and growth factors related with damage of SARS-CoV-2 spike protein by deactivating mitogen-activated protein kinase (MAPK)/NF-κB signaling in epithelial cells [237]. The nano-formulation of curcumin named “nanocurcumin” improved dissolution rate, saturation solubility, bioavailability, and drug stability. Tahmasebi et al. (2021) [238], in a randomized, double-blind-placebo controlled trial study, evidenced that the treatment with nanocurcumin led to an increase of the number of suppressor Treg cells and levels of transcription factor forkhead box protein P3 (FOXP3), IL10, IL35, and transforming growth factor-β (TGF-β). The same research group also demonstrated that nanocurcumin was able to reduce the frequency of Th17 cells and corresponding inflammatory factors in mild- and severe-COVID-19 patients [239].

## 4. Conclusions

It is now established that nutrition and nutritional supplements are an important role in the prevention and treatment of COVID-19 disease and post-COVID syndrome. Post-COVID syndrome refers to a variety of symptoms with a duration beyond the acute phase of COVID-19. It is mainly characterized by pulmonary, musculoskeletal, digestive, and neurological problems. It represents an emerging global crisis. However, the mechanisms by which SARS-CoV-2 may cause post-COVID syndrome and the best therapeutic options are not clearly defined. Besides the common therapies used for COVID-19, flavonoids, curcumin, melatonin, prebiotics, probiotics, and vitamin C, D, and E have shown encouraging data suggesting their use to prevent and counteract the symptoms of COVID-19 pandemic infection, and they are currently under study in the prevention and treatment of post-COVID syndrome as well. Nano-nutraceuticals may represent new strategies for the development of new therapies to curb COVID-19 and post-COVID syndrome. In any case, new studies are urgently needed to further investigate the molecular mechanisms played by nutraceuticals in the prevention and treatment of post-COVID syndrome. This will allow more rational and efficient use of these safe products.

## 5. Future Perspectives

Nutrition plays an important role in the management of the current COVID-19 and post-COVID syndrome. Numerous studies have demonstrated the importance of nutraceuticals for the prevention and treatment of these diseases. Future research may be addressed to in deep studies on the nutraceutical that have already demonstrated their usefulness and to new, potentially effective natural compounds. A longer efficacy with no side effects is a key requirement to plan future treatments needed to manage COVID-19 and post-COVID-19 infection. Specifically, in the last two years, efforts have been mainly focused on fighting COVID-19 in order to reduce hospitalizations and deaths. Now, is the time to focus on post-COVID, which is currently the most worrisome enemy and for which no validate therapeutic options are available.

## Figures and Tables

**Table 1:** RCTs studies regarding the use of vitamin C in the treatment of COVID-19 in relation to CRP, body temperature, lymphocyte counts, CD4+ T-cell counts, and P/F and SOFA scores.

Dose of Vitamin C	N° of Participants	Duration of Intervention	Outcome of Interest	Ref.
500 mg	120 hospitalized critically ill patients with COVID-19	14 days	A higher mean survival duration with respect to that of the control group (8 vs. 4 days, *p* < 0.01).	[48]
24 g	308 adults diagnosed with COVID-19 and transferred into ICUs	7 days	Ventilator-free days in the 28 days since admission to the ICU; Changes in SOFA scores, in plasma biomarkers of inflammation, and in pulmonary infection.	[89]
162.7 mg/kg for severe and 178.6 mg/kg for critical patients.	12 COVID-19 patients (six severe and six critical)	3 months	Improvement of CRP, body temperature, lymphocyte counts, CD4^+^ T-cell counts, and P/F and SOFA score.	[91]
100 mg/kg/day and a rate of 1 g/h for 7 days recovery	55 moderate COVID-19 patients	1 month	A shorter duration of SIRS (*p* = 0.0004); lower CRP levels (*p* = 0.005) and higher number of CD4^+^ T cells from Day 0 (on admission) to Day 7 (*p* = 0.04).	[91]
24 g	56 critical COVID-19 patients	7 days	Improvement in P/F ratio (*p* = 0.01); decline in IL-6 (*p* = 0.04).	[91]

CRP, C-reactive protein; ICU, intensive care unit; P/F, PaO_2_/FiO_2_; SIRS, systemic inflammatory response syndrome; SOFA, Sequential Organs Failure Assessment.

**Table 2:** RCTs studies of probiotic in the treatment of COVID-19 gastrointestinal symptoms.

Study Type	Study Subjects	Age Group	Number Enrolled	Intervention/Treatment	Primary Outcome Measures	Ref
Single-blind RCT	Patients with COVID-19	≥18 year	152	Oxygen–ozone therapy with dietary supplements SivoMixx	Number of patients, in treatment, needing orotracheal intubation.	[215]
RCT	COVID-19 patients requiring hospitalization	18–60 year	300	Combination of *Lactobacillus plantarum CECT7481*, *L. plantarum CECT 7484*, *L. plantarum CECT 7485*, and *Pediococcus acidilactici CECT 7483* vs. Placebo	Severity progression of COVID-19, stay at ICU, mortality ratio.	[216]
Double-blind RCT	Persons with household contact of COVID-19 patient	≥1 year	182	Probiotic (*Lactobacillus rhamnosus GG*) vs. Placebo	Changes in Shannon bacteria diversity.	[217]
Double-blind RCT	Healthcare workers without COVID-19	≥20 year	314	Probiotic (Lactobacillus) vs. Control (Maltodextrin)	Occurrence of SARS-CoV-2 infection in healthcare workers.	[218]
Open-label RCT	COVID-19 patients requiring hospitalization	≥18 year	40	Dietary Supplement: Probiotic vs. No intervention	Cases with discharge to ICU.	[219]
Double-blind RCT	COVID-19 patients with diarrhea	≥18 year	108	Synbiotic (Omnibiotic AAD: 2 *Bifidobacterium* strains, *Enterococcus*, 7 *Lactobacillus* strains) vs. Placebo	Duration of diarrhea.	[220]

SivoMixx: *Streptococcus thermophilus* DSM322245, *Bifidobacterium lactis* DSM32246, *Bifidobacterium lactis* DSM32247, *Lactobacillus acidophilus* DSM32241, *Lactobacillus helveticus* DSM32242, *Lactobacillus paracasei* DSM32243, *Lactobacillus plantarum* DSM32244, and *Lactobacillus brevis* DSM27961; ICU, intensive care unit; RCT, randomized controlled trial.

## Data Availability

Not applicable.

## Abbreviations

ACE2	Angiotensin-2 converting enzyme
Ang II	Angiotensin II
CARDS	COVID-19-associated acute respiratory distress syndrome
COVID-19	Coronavirus disease
CRP	C-reactive protein
HDIVC	High-Dose Intravenous Vitamin C
ICU	Intensive care unit
IL	Interleukin
NLRP3	Nod-like receptor family pyrin domain containing 3
RBD	Receptor-binding domain
RCT	Randomized controlled trial
SARS-CoV-2	Severe acute respiratory syndrome coronavirus 2
